# Impact of heat stress and hypercapnia on physiological, hematological, and behavioral profile of Tharparkar and Karan Fries heifers

**DOI:** 10.14202/vetworld.2017.1146-1155

**Published:** 2017-09-30

**Authors:** Priyanka Pandey, O. K. Hooda, Sunil Kumar

**Affiliations:** Animal Physiology Division, ICAR-National Dairy Research Institute, Karnal - 132 001, Haryana, India

**Keywords:** behavior, hematological, hypercapnia, hyperthermia, Tharparkar

## Abstract

**Aim:**

The present investigation was undertaken to study the impact of heat stress and hypercapnia on physiological, hematological, and behavioral profile of Tharparkar and Karan Fries (KF) heifers.

**Materials and Methods:**

The animals of both the breeds of Tharparkar and KF were exposed at different temperatures and CO_2_ levels. Exposure conditions of 25°C, 400 ppm CO_2_ level, and 60% relative humidity (RH) were taken as a control condition. The exposure conditions 40°C with two levels of CO_2_ 500 ppm and 600 ppm with RH 55±5% and exposure conditions 42°C with two levels of CO_2_ 500 ppm and 600 ppm with RH 55±5% were taken as treatments. The exposure period in each condition was 4 h daily for 5 consecutive days.

**Results:**

Physiological responses (respiration rate [RR], pulse rate [PR], and rectal temperature [RT]) were significantly (p<0.01) higher and different during all exposure conditions compared to control condition in both the breeds of cattle. KF heifers had higher RR, PR, and RT than Tharparkar heifers. Hematological parameters, namely, red blood cell, hemoglobin, and packed cell volume were significantly higher and different during all exposure condition than control in both the breeds, whereas no significant changes were observed in total leukocyte count and differential leukocyte count. Blood pH increased with increase in temperature and CO_2_ levels and was significantly higher than control conditions. PCO_2_ and base excess were significantly (p<0.05) lower, and PO_2_ was higher during different exposure conditions than control in both breeds. Restlessness and excitement signs were observed in all the exposure conditions as compared to control condition in both the breeds.

**Conclusion:**

Changes in physiological responses, behavioral pattern, and hematological parameters reflect the current functional status of the body system, and it can be used as an index for assessing the adaptation capacity of cattle to predict changes occurring in climate variables due to increasing CO_2_ levels and environmental temperature.

## Introduction

Climate change impacts on livestock are being witnessed all over the world, but its effects are more intense in Indian condition. Evidence from the Intergovernmental Panel on Climate Change [1] is now overwhelmingly convincing that climate change is real and it will become worse and the poorest will be affected more. Warming of global climate has multifaceted effects. Scientists have envisioned that increase in average global temperature above 1°C may be beyond the bearable limit of the present-day societies. Hot environment impairs production, reproduction, immune response, metabolic, and health status of livestock. Since the crossbred cattle are more sensitive to temperature rise than indigenous cattle, a rise of 2-6°C due to global warming will have more impact on growth, puberty, and maturity of crossbred cattle, and the negative effects of heat stress will become even more apparent in the future if climate change continues as predicted [2].

CO_2_ is an asphyxiant gas, the physiological effects of acute CO_2_ exposure are grouped together under the term hypercapnia, a subset of asphyxiation where breathing is too slow or shallow and causes respiratory acidosis while breathing too fast to the level of hyperventilation causes respiratory alkalosis. Blood performs a number of functions in the body, and any change in its constituents reflects the current functional status of the body system. Thus, the blood constituents can be used as an index for assessing the adaptation capacity of cattle to climate. McManus *et al*. [3] reported a strong positive correlation between packed cell volume (PCV) and hemoglobin (Hb) concentration indicating the significance of these parameters for heat tolerance in Brazilian sheep. No significant changes were observed in total leukocyte count (TLC) and total erythrocyte count (TEC) in Sahiwal and Karan Fries (KF) cattle exposed at 40°C and 45°C, but lymphocyte counts decreased, and neutrophil counts increased [4]. Bhan *et al*. [5] observed an increase in the mean TLC values when buffalo heifers were exposed to 42±1°C for 3 h in a climatic chamber. Insufficient ventilation, especially high CO_2_ to O_2_ ratio of animal’s house, stimulated respiration and circulation functions of animals [6]. Kadzere *et al*. [7] reported that PCO_2_ >40 mmHg stimulates respiration, whereas lower PCO_2_ inhibits it. This is because thermally induced hyperventilation decreases PCO_2_ [8].

The interactive effects of elevated CO_2_ and temperature stress under environmentally realistic scenarios of global climate change are not well understood in bovines, thereby hampering our ability to predict the consequences of global climate change on bovine physiology. In this study, the short-term exposure (5 days) of Tharparkar and KF heifers at different temperatures and CO_2_ levels corresponding to the present-day conditions and the predicted future changes in global climate change has been reported.

## Materials and Methods

### Ethical approval

Experiment was approved by the Institutional Animal Ethics Committee constituted as per the article no.13 of the CPCSEA rules, laid down by Government of India.

### Study area

The National Dairy Research Institute (NDRI), Karnal, is situated at an altitude of 250 meter above mean sea level. Latitude and longitude positions being 29°42″ N and 79°54″ E, respectively.

### Experimental animals

Twelve apparently healthy heifers, six each of Tharparkar and KF varying from 1 to 2 years of age and average body weight of 196±3.05 and 196.16±2.21 kg, respectively, were taken from the NDRI, Karnal. The experimental animals were maintained and fed as per the standard practice followed at the herd of the NDRI, Karnal. The animals were offered a ration consisting of concentrate mixture and roughages (berseem, maize, or jowar as per the availability at the farm). Concentrate mixture (crude protein 19.81% and total digestible nutrients 70%) contained maize 33%, groundnut cake (oiled) 21%, mustard oil cake (oiled) 12%, wheat bran 20%, de oiled rice bran 11%, mineral mixture 2%, and common salt 1%. Fresh tap water was made available for drinking throughout the time to all the animals throughout the experiment.

### Experimental protocol

- Animals of both breeds were exposed in a climatic chamber at temperature 25°C, CO_2_ level 400 ppm, and relative humidity (RH) 60% 4 h daily for 5 consecutive days and served as control.
- Animals of both breeds were exposed in a climatic chamber at temperature 40°C, CO_2_ level 500 ppm, and RH 55±5% 4 h daily for 5 consecutive days. After 14 days rest, all the animals were again exposed at temperature 40°C, CO_2_ level 600 ppm, and RH 55±5% 4 h daily for 5 consecutive days.
- After 21 days rest, the animals of both breeds were exposed at temperature 42°C, CO_2_ levels 500 and 600 ppm, and RH 55±5% in the same way as in B.

### Recording of physiological responses

Physiological responses (respiration rate [RR], pulse rate [PR], and rectal temperature [RT]) were recorded before exposure and at the end of 5^th^ day experiment, and for these routine methods were followed. Animal behavior was recorded by the CCTV camera during the exposure period.

### Collection of blood samples

Blood samples from each animal were taken from jugular vein in heparin-coated vacutainer tubes before exposure and at the end of 5^th^ day exposure in all exposure conditions. From this, approximately 1.0 ml of blood of each animal was withdrawn in a 1.0 ml syringe, and the syringe was made an airtight so that no air can enter into the syringe. This portion of blood was used immediately for blood gas analysis and base excess (BE) determination in Nova Biochemical blood gas analyzer and blood hematological studies in BC-2800 vet blood autoanalyzer.

### Statistical analysis

Data were analyzed using one-way analysis of variance by Statistical Analysis System [9] Software Program, version 9.1, and the results were expressed as a mean ± standard error and considered statistically significant at 1% and 5% level.

## Results and Discussion

### Physiological responses

The mean values of physiological responses, i.e., RR, PR, and RT of Tharparkar and KF heifers during control as well as different exposure conditions are given in Table-1. The RR, PR, and RT of both breeds were significantly higher (p<0.01) at 40°C, 500 and 600 ppm CO_2_; 42°C, 500 and 600 ppm CO_2_ compared to control conditions, and the increase in these parameters was more in KF than Tharparkar.

**Table-1:** Effect of elevated temperature and CO_2_ levels on physiological responses of Tharparkar and Karan Fries heifers.

Parameter	Breed	25°C (control)	40°C		42°C	
		
		CO_2_ levels (ppm)	CO_2_ levels (ppm)		CO_2_ levels (ppm)	
		
		400	500	600	500	600
RR (breaths/min)	Tharparkar	22.66^Ae^±0.42	60.66^Bd^±0.33	64.83^Bc^±0.73	67.08^Bb^±0.60	69.25^Ba^±0.52
	Karan Fries	23.83^Ae^±0.60	78.0^Ad^±0.73	81.33^Ac^±0.55	86.0^Ab^±0.85	90.16^Aa^±0.47
PR (beats/min)	Tharparkar	68.16^Ad^±0.30	74.16^Bc^±0.87	77.0^Bb^±0.81	78.5^Bb^±0.42	82.0^Ba^±0.73
	Karan Fries	69.83^Ad^±0.60	77.16^Ac^±0.60	79.5^Ac^±0.76	82.5^Ab^±0.76	86.16^Aa^±0.40
RT (°F)	Tharparkar	100.8^Ad^±0.26	102.48^Ac^±0.12	102.86^Bcb^±0.16	103.30^Bb^±0.20	104.38^Ba^±0.16
	Karan Fries	101.2^Ad^±0.20	102.80^Ac^±0.16	103.58^Ab^±0.20	104.21^Ab^±0.07	105.68^Aa^±0.15

Mean with different superscripts (A and B) in column differs significantly between the breeds for respective parameter for each exposure condition. Mean with different superscripts (a, b, c, d, and e) in the same row differs significantly for respective breed for each exposure condition. RR=Respiration rate, PR=Pulse rate, RT=Rectal temperature

Increased RR is the first reaction when animals are exposed to an environmental temperature above thermoneutral zone. The significance of this increased RR under heat stress is that it enables the animals to dissipate the excess of the body heat by respiratory evaporative cooling through the expired air, which accounts for 30% of the total heat dissipation from the body [10]. Davinder [11] reported significantly (p<0.01) higher RR in KF and Murrah calves during summer than winter season. Similar results were reported by Indu [12] in Tharparkar and KF calves when the calves were exposed at 44°C for 4 h in a climatic chamber. Das *et al*. [13] reported that air temperatures above 20-25°C in temperate climate and 25-37°C in a tropical climate such as in India, enhance heat gain beyond that lost from the body and induce HS which results into increased body surface temperature, RR, heart rate, and RT which leads to decrease in feed intake, production, and reproductive efficiency of animals. Similar findings were reported in Holstein Friesian and Brown Swiss cattle when cows were exposed to 0.45% of CO_2_ level [6]. The differences in RR were nonsignificant at control condition, but at all other exposure conditions, the RR of KF calves was significantly higher than Tharparkar. It might be due to low heat tolerance of KF compared to Tharparkar. The findings of the present study are also in support of those reported by Singh and Upadhyay [14] who also observed higher RR in KF than Sahiwal cattle during heat stress.

Hooda and Upadhyay [15] reported that the pulse rate reflects primarily the homeostasis of circulation along with the general metabolic status. The observed accelerated PR could be due to the redistribution of blood to peripheral tissues to give a chance for more heat to be lost by sensible and insensible means during exercise and exposure to heat stress. Mohr *et al*. [16] reported that the mean resting PR of healthy animals increased from 105±11.1 to 114±12.5 bpm under the influence of external stress and sickness. On increasing the CO_2_ levels from 400 to 500 and 600 ppm, there was a significant increase in PR in both the breeds. Similar findings were reported in Holstein Friesian and Brown Swiss cattle which were exposed to 0.45% of CO_2_ level [6]. The authors suggested that a high CO_2_ level stimulates the circulatory function of the animal which might have resulted in increased PR of the animals. Sagsoz *et al*. [17] also observed increased PR of Holstein Friesian and Brown Swiss cattle from 65.9 to 69.4 and 61.8 to 62.4 beats/minute, respectively, when the cows were exposed to 522.9 ppm of CO_2_ level, 21°C temperature, and 82% RH. PR was significantly higher in KF compared to Tharparkar cattle in all the exposure conditions which might be an acclimation process to dissipate extra heat by this breed. Gaughan *et al*. [18] reported that there was a greater engagement of breeds for heat loss mechanisms which are less adapted to a climate. Aggarwal and Upadhyay [19] also observed similar changes in PR and reported that PR increased from 61 to 79 and 67 to 86 beats/minute in Sahiwal and crossbred cattle, respectively, after 4 h of solar radiation exposure.

Silanikove [20] stated that RT is an indicator of thermal balance and may be effective in quantifying the discomfort level of the animals during thermal stress. Moran [21] reported that RT of buffaloes was easily influenced by their surrounding temperature, and it increased rapidly during exposures in a hot climate. Chandra *et al*. [22] reported that RT of KF remained significantly high compared to Sahiwal and Tharparkar cattle during different seasons and the magnitude of increase in RT was higher in KF than Sahiwal and Tharparkar during summer season. Randhawa *et al*. [23] reported that the temperature humidity index (THI) describes the effect of environment on animal’s ability to dissipate heat. During drought, the ambient temperature and RH frequently exceed the critical comfort level of THI (72), resulting in elevated body temperature and panting. In this study, RT was significantly higher in KF compared to Tharparkar at different conditions of elevated temperature and CO_2_ levels. The higher RT in *Bos taurus* than in *Bos indicus* cattle and attributed it to notable differences among different breeds of cattle in their ability to regulate RT. Hansen [24] also stated that zebu cattle are better in the regulation of their body temperature than those of *B. taurus* cattle of European origin in response to heat stress. RT was significantly different and higher at elevated CO_2_ levels and high exposure temperatures in both the breeds. It has been observed that, at elevated CO_2_ levels, the condition of hypoxia is generated and acute hypoxia causes a reduction in body temperature. However, in this study, there was an increase in RT which might be due to high temperature and interactive effects of elevated CO_2_ level in different exposure conditions.

### Hematological parameter

The mean values of red blood cell (RBC), hematocrit, and Hb concentration in Tharparkar and KF heifers are given in Table-2. The concentration of TEC hematocrit and Hb in both breeds increased with increase in temperature and CO_2_ levels, and the values were significantly different at different exposure conditions. Between the breeds, RBC, hematocrit, and Hb were significantly higher in KF than Tharparkar at 40°C and 500 and 600 ppm CO_2_; 42°C and 500 and 600 ppm CO_2_ levels.

**Table-2:** Effect of elevated temperature and CO_2_ levels on blood parameters of Tharparkar and Karan Fries heifers.

Parameter	Breed	25°C (control)	40°C		42°C	
		
		CO_2_ levels (ppm)	CO_2_ levels (ppm)		CO_2_ levels (ppm)	
		
		400	500	600	500	600
Red blood cells (×10^6^/µl)	Tharparkar	7.23^Ad^±0.18	7.9^Adc^±0.22	8.53^Bbc^±0.17	8.8^Bba^±0.18	9.6^Ba^±0.17
	Karan Fries	7.58^Ae^±0.08	8.3^Ad^±0.12	9.16^Ac^±0.16	9.9^Ab^±0.19	10.62^Ba^±0.19
Hemoglobin (g/dl)	Tharparkar	9.1^Ae^±0.14	10.46^Bd^±0.13	11.18^Bc^±0.05	12.3^Bb^±0.13	13.26^Ba^±0.09
	Karan Fries	9.4^Ae^±0.16	10.85^Ad^±0.11	11.80^Ac^±0.10	12.83^Ab^±0.10	14.33^Aa^±0.19
Hematocrit (%)	Tharparkar	31.66^Bc^±0.05	33.21^Ab^±0.18	34.28^Bb^±0.18	36.31^Ba^±0.32	37.03^Ba^±0.42
	Karan Fries	32.04^Ae^±0.15	33.93^Bd^±0.25	35.81^Ac^±0.21	37.8^Ab^±0.24	38.8^Aa^±0.24
White blood cells (×10^3^/µl)	Tharparkar	13.44±0.38	13.72±0.52	13.74±0.72	13.34±0.61	13.88±0.73
	Karan Fries	12.97±0.58	12.76±0.62	12.94±0.35	12.62±0.29	13.2±0.40
Lymphocyte (%)	Tharparkar	61.54±0.13	61.06±0.15	61.21±0.28	61.28±0.30	61.30±0.29
	Karan Fries	62.52±0.32	62.42±0.22	62.45±0.24	62.79±0.40	62.82±0.42
Granulocyte (%)	Tharparkar	22.68±0.39	23.46±1.10	24.68±1.12	25.82±1.13	23.74±1.39
	Karan Fries	22.89±1.49	22.18±1.26	23.6±1.10	25.86±1.37	24.86±1.37
Monocyte (%)	Tharparkar	13.16±1.57	13.99±1.60	14.68±1.18	13.48±1.16	12.56±1.76
	Karan Fries	14.73±1.25	14.98±1.08	15.54±0.30	14.38±0.66	12.94±0.66

Mean with different superscripts (A and B) in column differs significantly between the breeds for respective parameter for each exposure condition. Mean with different superscripts (a, b, c, d, and e) in the same row differ significantly for respective breed for each exposure condition

Lateef *et al*. [25] reported the higher mean value of TEC in Kankrej cattle during hot summer. The authors suggested that the increase in RBC counts might be due to hemoconcentration during stressful condition and also due to higher PCV. In this study, hypercapnia (elevated level of CO_2_) resulted in increase of RBC counts is in support of Abdelatif *et al*. [26] who reported increase in RBC count and attributed that the increase in RBC count might be due to hypoxia-dependent upregulation of erythropoietin and also due to age, temperature, and seasonal conditions.

Shandya *et al*. [27] reported that heat stress caused an increase in PCV in buffaloes and attributed it to the loss of water from body due to dehydration. Similar observations were recorded by Omran *et al*. [28]. The author suggested that, when animals were exposed to high ambient temperatures, hemoconcentration developed due to dehydration, asphyxia, or excitement, which caused the release of erythrocytes from spleen, and thereby resulted in abnormally higher PCV levels. The higher PCV values might have caused due to increase in RBC and Hb concentration. At a higher level of CO_2_, there was a significant increase in Hb concentration in both Tharparkar and KF breeds of cattle.

The mean values of TLC, lymphocyte, granulocyte, and monocyte of Tharparkar and KF heifers during control and different exposure conditions are given in Table-2. In this study, it was observed that there was no significant difference in the TLC, lymphocyte, granulocyte, and monocyte count in both breeds at different temperatures and CO_2_ levels compared to control conditions. Similar results were reported in calves [29]. In both, the breeds, the leukocyte concentrations at 25°C, 400 ppm CO_2_; 40°C, 500 and 600 ppm CO_2_; and 42°C, 500 and 600 ppm CO_2_ levels were non-significant statistically. Haque *et al*. [30] observed no change in granulocytes in young and adult Murrah buffaloes when they were exposed at 40°C, 42°C, and 45°C in climatic chamber for 4 h. Mirzadeh *et al*. [31] also reported lower levels of white blood cell in different breeds of cattle during summer than spring.

### Blood gases

The values of blood pH, PCO_2_, and PO_2_ in Tharparkar and KF heifers are given in Table-3. The blood pH and PO_2_ increased with increase in temperature and CO_2_ levels in both breeds, whereas the PCO_2_ decreased with the increase in temperature and CO_2_ levels and were significantly different in different exposure conditions in both Tharparkar and KF heifers. Between breeds, the values of blood pH were observed significantly (p<0.05) difference only at 42°C and 600 ppm CO_2_ level and PCO_2_ were significantly lower in KF than Tharparkar heifers at all exposure conditions, whereas the levels of PO_2_ were non-significant statistically at all exposure conditions.

**Table-3:** Effect of elevated temperature and CO_2_ levels in on blood pH and blood gases of Tharparkar and Karan Fries heifers.

Parameters	Breed	25°C (control)	40°C		42°C	
		
		CO_2_ levels (ppm)	CO_2_ levels (ppm)		CO_2_ levels (ppm)	
		
		400	500	600	500	600
pH	Tharparkar	7.33^Ae^±0.01	7.36^Ad^±0.005	7.39^Ac^±0.005	7.42^Ab^±0.004	7.45^Ba^±0.005
	Karan Fries	7.32^Ae^±0.01	7.37^Ad^±0.005	7.40^Ac^±0.004	7.44^Ab^±0.003	7.47^Aa^±0.003
PCO_2_ (mmHg)	Tharparkar	55.03^Aa^±0.22	53.16^Ab^±0.07	50.83^Ac^±0.25	46.21^Ad^±0.13	42.13^Ae^±0.22
	Karan Fries	54.31^Aa^±0.27	51.3^Bb^±0.34	48.11^Bc^±0.26	43.36^Bd^±0.28	39.75^Be^±0.19
PO_2_ (mmHg)	Tharparkar	41.88^Ad^±0.57	46.78^Ac^±0.63	48.81^Abc^±0.57	51.31^Aba^±0.65	53.33^Aa^±0.63
	Karan Fries	40.50^Be^±0.12	46.36^Ad^±0.33	48.4^Ac^±0.33	50.41^Ab^±0.46	54.38^Aa^±0.26

Mean with different superscripts (A and B) in column differs significantly between the breeds for respective parameter for each exposure condition. Mean with different superscripts (a, b, c, d, and e) in the same row differ significantly for respective breed for each exposure condition

The observation of blood pH is in accordance with the findings of Schneider *et al*. [32] who reported that cow subjected to heat stress had higher blood pH during hot hours compared to thermoneutral environment. Sivakumar *et al*. [33] reported increased pH level in goats under heat stress. Kadzere *et al*. [7] reported that PCO_2_ >40 mm Hg stimulates RR, whereas lower PCO_2_ inhibits it. Helal *et al*. [34] found that increasing the concentration of inhaled CO_2_ by breathing is associated with hyperventilation and decrease in blood PCO_2_. The decrease in blood PCO_2_ is due to thermally induced hyperventilation which is similar to the finding of Sivakumar *et al*. [33].

The findings of PO_2_ in the present study are in accordance with the findings of Hales and Findley [35] who reported that heat stress raised the PO_2_ and the increase was attributed to increase in alveolar ventilation. Wojtas *et al*. [36] also reported an increased level of PO_2_ in Polish Merino sheep when expose to high temperature (30°C). The increase in PO_2_ in the present study due to elevated CO_2_ levels is in accordance with the findings of Sabuncuoglu *et al*. [6] who reported that in cattle blood PO_2_ increased by higher temperature, higher CO_2_ level, and RH in cattle.

### BE

**Figure-1:**
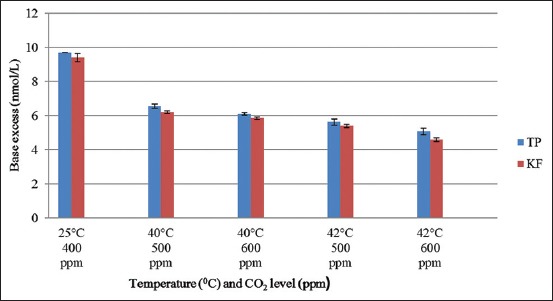
Effect of elevated temperature and CO_2_ levels on the base excess of Tharparkar and Karan Fries heifers.

The value of blood BE was significantly (*P*<0.01) lower during different exposure conditions compared to control conditions in both Tharparkar and KF heifers (Figure-1).

The findings are in accordance with the result of Srikandakumar *et al*. [37] who reported a decrease in BE in sheep when subjected to heat stress. A similar finding was reported by Hussein and Aamer [38] in ovine. The probable reason of reduction in BE might be due to low oxygen tension (PO_2_) which might have increased anaerobic glycolysis to cause excess production of lactic acid. Korde *et al*. [39] also reported low BE in buffalo calves in heat stress condition and suggested that it might be a compensatory response to respiratory alkalosis. West *et al*. [40] also observed low BE in heat-stressed dairy cows and suggested that the reduction in BE might be due to either increased respiratory rate/panting under heat stress, which eliminated CO_2_ in excess and caused decreased PCO_2_ as well as carbonic acid, or it might be due to decrease in the non-bicarbonate buffer system, particularly Hb which resulted in decreased levels of buffer in blood.

### Animal behavior

The parameters of physiological behavior in Tharparkar and KF are given in Table-4. During control conditions, both the breeds of cattle were calm and quiet with all the physiological parameters in the normal range. During exposure, there were significant (p<0.05) changes in behavioral parameters of both breeds as given in Table-4. Moreover, changes were more apparent in KF compare to Tharparkar.

**Table-4:** Effect of elevated temperature and CO_2_ levels on behavior of Tharparkar and Karan Fries heifers.

Parameter	Breed	25°C (control)	40°C		42°C	
		
		CO_2_ levels (ppm)	CO_2_ levels (ppm)		CO_2_ levels (ppm)	
		
		400	500	600	500	600
Restlessness	Tharparkar	Calm and quiet	Disturbed	Bad temperament	Excited	Furious
	Karan Fries	Calm and quiet	More disturbed	Bad temperament	Highly excited	More furious
Open mouth panting	Tharparkar	Absent	High	High	Higher	Higher
	Karan Fries	Absent	Higher	Higher	Highest	Highest
Tongue protrusion	Tharparkar	Absent	Occasional mouth opening	Frequent appearance of tongue	Continuous protrusion	Continuous protrusion
	Karan Fries	Absent	Frequent appearance of tongue	Continuous protrusion	Tongue fully out	Tongue fully out
Frothy salivation	Tharparkar	Absent	Dribbling of saliva starts	Continuous dribbling	Appearance of froth on upper lip	Full mouth frothing
	Karan Fries	Absent	Dribbling of saliva starts	Continuous dribbling	Appearance of froth on upper lip	Full mouth frothing
Muzzle secretion	Tharparkar	Absent	High	High	Higher	Higher
	Karan Fries	Absent	Higher	Higher	Highest	Highest
Standing time	Tharparkar	Less	Increased	Increased	Highly increased	Highly increased
	Karan Fries	Less	Increased	Increased	Highly increased	Highly increased

Behavior of both the breeds changed with changes in temperature and CO_2_ levels. Animals of both breeds were more nervous and restlessness, and there was increased salivation, profuse tongue protrusion, and panting. Panting was observed when the normal heat dissipation mechanism was compromised, and it culminated into evaporative cooling as the most effective means of heat loss. All the physiological parameters increased compared to control conditions. Both the breeds spent most of the time in standing with the increase in stress level due to increased temperature and CO_2_ levels. Water consumption increased in both the breeds. The changes were higher in KF than Tharparkar heifers indicating that KF were more susceptible to heat stress. Nardone *et al*. [41] reported increased panting, lowered saliva production, and decreased rumination during thermal stress in livestock. One of the changes associated with behavior during heat load is an increase in time spent in standing [42]. Cattle may reduce the effects of high heat load by increasing water consumption. Access to cool drinking water improved weight gain in feedlot cattle in summer [43]. The behavioral changes help in the acclimatization process of animals during a stressful period and can be used to assess the impact of thermal stress in ruminants.

## Conclusion

On the basis of this study, it can be concluded that the fluctuations in physiological responses, blood constituents, and behavioral changes during heat stress and hypercapnia are an effort to maintain normal homeostasis of the body and can be used as an index for assessing the adaptation capacity of cattle to changing climate that helps in the acclimatization process of animals during stressful period and can be used to assess the impact of thermal and CO_2_ stress in ruminants.

## Authors’ Contributions

OKH designed the study. PP conducted the experiment. PP and SK performed statistical analysis. PP and SK drafted and revised the manuscript under the guidance of OKH. All authors read and approved the final manuscript.
